# Reference charts for first‐trimester placental three‐dimensional fractional moving blood volume derived using OxNNet

**DOI:** 10.1002/uog.70161

**Published:** 2026-01-07

**Authors:** S. Mathewlynn, L. N. Starck, Y. Yin, M. Soltaninejad, M. Swinburne, K. H. Nicolaides, A. Syngelaki, A. G. Contreras, S. Bigiotti, E.‐M. Woess, S. Gerry, S. Collins

**Affiliations:** ^1^ Nuffield Department of Women's and Reproductive Health University of Oxford Oxford UK; ^2^ Oxford University Hospitals NHS Foundation Trust Oxford UK; ^3^ Fetal Medicine Research Institute, King's College Hospital London UK; ^4^ University of Bristol Bristol UK; ^5^ Centre for Statistics in Medicine, Nuffield Department of Orthopaedics, Rheumatology and Musculoskeletal Sciences University of Oxford Oxford UK; ^6^ Birmingham Women and Children's NHS Foundation Trust Birmingham UK

**Keywords:** biomarker, deep learning, fetal growth restriction, first‐trimester pregnancy, fractional moving blood volume, placenta, power Doppler ultrasonography, prenatal ultrasonography, pre‐eclampsia, reference values

## Abstract

**Objective:**

To establish a comprehensive reference range for first‐trimester placental three‐dimensional (3D) single‐vessel fractional moving blood volume (svFMBV) using the OxNNet toolkit, based on values observed in healthy pregnancies.

**Methods:**

This study utilized data from the First‐trimester Placental Ultrasound (FirstPLUS) study, a longitudinal observational cohort study conducted between March and November 2022 at King's College Hospital, London, UK. Participants underwent 3D ultrasound assessment of the placenta, including power Doppler imaging, during routine first‐trimester screening. The OxNNet toolkit was used for automated placental segmentation and 3D‐svFMBV calculation. Quality control was performed in three stages to ensure image completeness and segmentation accuracy. Quantile regression and lambda‐mu‐sigma (LMS) modeling were used to construct reference charts for 3D‐svFMBV. Model fit was assessed using the Akaike information criterion, and centile curves were constructed.

**Results:**

The final cohort comprised 2547 cases. Visual assessment of histograms and quantile−quantile plots revealed positive skew in 3D‐svFMBV determined at five specific locations within the uteroplacental vasculature. LMS modeling provided the best fit for constructing centile charts, with the Box–Cox Power Exponential original distribution used in most cases. The resulting centile charts demonstrated close agreement between predicted and observed centiles, with minimal deviation across all target centiles.

**Conclusions:**

This study provides novel reference ranges for first‐trimester placental 3D‐svFMBV at five locations within the uteroplacental vasculature. These findings offer a valuable foundation for future research into placental function and pregnancy outcome. © 2026 The Author(s). *Ultrasound in Obstetrics & Gynecology* published by John Wiley & Sons Ltd on behalf of International Society of Ultrasound in Obstetrics and Gynecology.

## INTRODUCTION

Placental disease, including fetal growth restriction (FGR) and pre‐eclampsia (PE), contributes significantly to perinatal morbidity and mortality^1^, ^2^, ^3^, ^4^. Early risk stratification, enhanced surveillance and low‐dose aspirin prophylaxis are the cornerstones of current mitigation efforts^5^, ^6^. Novel biomarkers with the potential to improve first‐trimester screening are therefore of considerable interest, and first‐trimester three‐dimensional (3D) placental ultrasonography offers potential in this regard.

**Figure 1:**
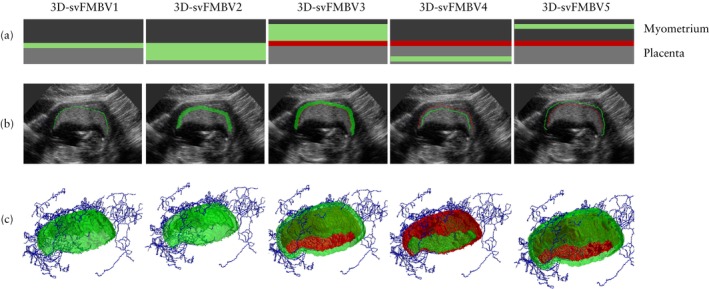
Schematic representations (a), two‐dimensional ultrasound images (b) and three‐dimensional renderings (c) of five target locations within uteroplacental vasculature at which three‐dimensional single‐vessel fractional moving blood volume (3D‐svFMBV) was assessed. Green indicates FMBV target area, red indicates uteroplacental interface and blue is skeletonized center line of blood vessels.

**Table 1:** Definitions of three‐dimensional single‐vessel fractional moving blood volume (3D‐svFMBV) used in this study

Identifier	Location	Target
3D‐svFMBV1	1‐mm thickness from UPI into placenta	UPI
3D‐svFMBV2	5‐mm thickness from UPI into placenta	IVS
3D‐svFMBV3	5‐mm thickness from UPI into myometrium	MVP
3D‐svFMBV4	1‐mm thickness at 5‐mm distance from UPI into placenta	Distal IVS
3D‐svFMBV5	1‐mm thickness at 5‐mm distance from UPI into myometrium	Proximal MVP

IVS, intervillous space; MVP, myometrial vascular plexus; UPI, uteroplacental interface.

The OxNNet toolkit is a fully convolutional neural network developed at the University of Oxford, Oxford, UK. It uses deep learning to achieve fully automated segmentation of the placenta, including the uteroplacental interface (UPI), as well as the amniotic fluid and fetus from 3D ultrasound images^5^, ^6^. Placental segmentation is a critical step in the calculation of placental 3D fractional moving blood volume (FMBV), a validated measure of perfusion derived from the power Doppler signal, as first described by Rubin *et al*.^7^, ^8^, ^9^. Power Doppler is less affected by velocity, flow direction and angle of insonation compared with color Doppler, making it ideal for assessing perfusion through the slow‐flowing and convoluted myometrial vascular plexus^10^, ^11^, ^12^. However, interpatient comparisons are confounded by tissue‐dependent signal attenuation, necessitating calibration against a reference point of presumed 100% signal amplitude. Rubin *et al*.^8^ extrapolated this value from a region of interest using cumulative signal curves, but its accuracy is limited by vessel size, background noise and the absence of a clear ‘knee’ point in highly vascular tissues^13^. OxNNet overcomes these limitations by automatically selecting a single reference vessel (a process discussed in detail by Yin *et al*.^13^), enabling calculation of single‐vessel 3D‐FMBV (3D‐svFMBV). 3D‐svFMBV can be assessed at various locations within the myometrial vascular plexus and the placenta in relation to the reference point of the UPI (Figure 1, Table 1).

Reduced vascularity at the UPI and intervillous space has been observed in pregnancies that develop PE, but not in normotensive small‐for‐gestational‐age pregnancies^14^. Therefore, it is possible that 3D‐svFMBV could help to differentiate between these phenotypes.

Our aim was to establish a comprehensive reference range for OxNNet‐derived first‐trimester placental 3D‐svFMBV based on healthy pregnancies. This reference range should provide a foundation for future research and facilitate the exploration of 3D‐svFMBV as a potential screening biomarker in prenatal care.

## METHODS

### Study design

This research utilized data from the First‐trimester Placental Ultrasound (FirstPLUS) study, a longitudinal observational cohort study of pregnant individuals undergoing first‐trimester screening between March and November 2022 at the Harris Birthright Research Centre for Fetal Medicine, King's College Hospital NHS Foundation Trust, London, UK. Participants were recruited at the time of their routine ultrasound assessment, conducted between 11 + 2 and 14 + 1 weeks' gestation. In addition to standard care, a 3D ultrasound scan of the placenta was performed, including power Doppler imaging. After the single study visit, participants continued to receive routine maternity care, and pregnancy outcomes were extracted subsequently from electronic patient records.

This study adhered to the Strengthening the Reporting of Observational Studies in Epidemiology (STROBE) guidelines^15^. The West Midlands–Solihull Research Ethics Committee approved the FirstPLUS study on 8 March 2022 (reference 22/WM/0039). Participants provided written informed consent and could withdraw from the study at any time.

### Study population

Participants were approached during their first‐trimester combined test. Inclusion criteria were: singleton pregnancy; age ≥ 18 years; attending for first‐trimester screening between 11 + 2 and 14 + 1 weeks' gestation; able to understand English or to access appropriate methods of translation; and deemed able to participate in study activities in the opinion of the investigators. Exclusion criteria were: multiple pregnancy; non‐viable pregnancy or pregnancy of uncertain viability; major fetal defect found at the scan; chromosomal abnormality discovered later in pregnancy; or incomplete combined test (e.g. unable to measure nuchal translucency).

To construct the reference charts, we analyzed a subgroup of the FirstPLUS cohort. The objective was to examine a group in which the placental vasculature was unlikely to be influenced by pathology, thereby producing a proscriptive reference (reflecting the range of values observed in a healthy population) rather than a descriptive reference (reflecting the entire population, including those with pathology). Specifically, our aim was to establish the normal range of placental 3D‐svFMBV in apparently healthy pregnancies. Consequently, we excluded cases meeting any of the following criteria: miscarriage, stillbirth, termination of pregnancy, neonatal death, preterm birth, major fetal anomaly identified subsequent to the research scan, evidence of FGR, neonatal unit admission, maternal diabetes mellitus (pre‐existing or gestational), maternal hypertensive disease (chronic hypertension, gestational hypertension or PE), antiphospholipid syndrome or systemic lupus erythematosus. Cases with incomplete outcome data were also excluded.

### Definitions

FGR was defined as a birth weight < 3^rd^ centile, or a birth weight < 10^th^ centile accompanied by a confirmed Doppler abnormality at the time of the final ultrasound scan. A Doppler abnormality was defined as an umbilical artery pulsatility index > 95^th^ centile or a cerebroplacental ratio < 5^th^ centile at the final ultrasound assessment prior to birth, in accordance with the reference ranges of Ciobanu *et al*.^16^. Birth‐weight centiles were calculated using the Fetal Medicine Foundation (FMF) population birth‐weight charts^17^. PE and gestational hypertension were defined according to the guideline of the American College of Obstetricians and Gynecologists (ACOG)^18^. Gestational diabetes mellitus was diagnosed using an oral glucose tolerance test, following the timing and diagnostic thresholds recommended by the National Institute for Health and Care Excellence (NICE)^19^. Preterm birth was defined as delivery before  37 + 0 weeks. Stillbirth was defined as birth without signs of life ≥ 24 + 0 weeks. Miscarriage was defined as ultrasound‐confirmed *in‐utero* fetal demise, or birth without signs of life, ≤ 23 + 6 weeks.

### Placental ultrasonography

Ultrasound examinations were conducted using GE Voluson™ E6 or E8 ultrasound machines (GE Healthcare, Zipf, Austria), equipped with a RAB4‐8‐D 3D/4D curved‐array abdominal transducer (4–8.5 MHz), and were performed by suitably trained fetal medicine fellows. The ultrasound equipment was preconfigured with the appropriate settings, which were saved as a preset mode to ensure consistency (detailed settings are provided in Table S1). After viability of the pregnancy had been confirmed, crown–rump length (CRL) and nuchal translucency were measured in accordance with standard protocols. Pregnancy dating was based on the CRL measurement^20^, except in cases involving assisted reproduction, for which the date of embryo transfer was used.

The placental ultrasound scan commenced with determining the optimal probe placement for 3D imaging of the entire placenta, which was typically a cross‐sectional plane near the center of the placenta. Grayscale gain settings were calibrated to ensure clear differentiation of the placenta from surrounding tissues on visual assessment. Power Doppler gain settings were finely tuned to obtain the individual's sub‐noise gain setting: the gain was increased until flooding of the image occurred, and then gradually reduced until the noise was just eliminated and no further^21^, ^22^. A volume was captured to assess for completeness of the placenta and underlying myometrium, and for the presence of power Doppler flash artifacts. Volume capture was repeated if the placenta was deemed to be incomplete or if more than five power Doppler flash artifacts were present.

### Image processing

Images were exported from ultrasound machines in Kretzfile 1.0 format (*.vol extension) without compression and were analyzed retrospectively by the Placental Imaging Research Group, Nuffield Department of Women's and Reproductive Health, University of Oxford, Oxford, UK. The placenta was segmented using the OxNNet toolkit, and 3D‐svFMBV was calculated as described previously^5^, ^6^, ^13^, ^23^. The power Doppler signal was calibrated and 3D‐svFMBV was calculated using the method described by Yin *et al*.^13^. A three‐stage quality control (QC) process was then undertaken.

#### Quality control: Stage 1

Before placental segmentation, trained sonographers reviewed all of the images for completeness. Images were excluded if the placenta appeared incomplete, the machine settings were incorrect, there was excessive power Doppler artifact (more than five flash artifacts intersecting with the placenta) or image quality was too poor for a human operator to delineate the placental contour.

#### Quality control: Stage 2

In the second stage of QC, placental and amniotic fluid segmentation were performed using both the current and original iterations of OxNNet. The segmentation obtained by each method was compared using the Dice similarity coefficient. Cases with poor agreement were reviewed manually, and those with undeterminable placental contours were excluded owing to poor image quality. Images were identified for review in three rounds: (1) agreement of placental segmentation < 60%, irrespective of agreement of amniotic fluid segmentation; (2) agreement of placental segmentation < 75% and agreement of amniotic fluid segmentation < 40%; and (3) agreement of amniotic fluid segmentation < 40%, irrespective of agreement of placental segmentation.

#### Quality control: Stage 3

In the third stage of QC, outliers were identified using the interquartile range (IQR) method for each day of gestation. For each day of gestation, the first (Q1) and third (Q3) quartiles of placental volume measurement were calculated. Measurements below Q1 − (1.5 × IQR) or above Q3 + (1.5 × IQR) were considered outliers. Outliers were reviewed manually by S.M. and M.So. to assess image quality and segmentation accuracy. Outliers were excluded if the image quality was insufficient to reliably determine the correct placental contour.

### Statistical analysis

Statistical analysis was conducted using R version 4.3.1 and RStudio version 2024.09.0 + 375 (R Foundation for Statistical Computing Platform, Vienna, Austria).

#### Sample size estimation

The sample size of the FirstPLUS study was predetermined to gather sufficient data to construct multivariable predictive models, considering attrition and loss to follow‐up. Using the method of Riley *et al*.^24^ and the *pmsampsize* version 1.1.3 package in R^25^, we calculated that, with a 2% incidence rate, 20 parameters and a C‐statistic of 0.9, 2171 cases would be sufficient. For a C‐statistic of 0.8, 2992 cases would be needed. Thus, a sample size of 4000 cases was selected as a pragmatic recruitment target.

To evaluate whether the available cases (after exclusions) were adequate for constructing reference charts, we calculated the precision of the data in estimating specific centiles (2.5^th^ and 97.5^th^), using the following formula^26^:

$${\mathrm{SE}}_{p}=\mathrm{SD}\sqrt{\left(1+\frac{1}{2}{Z_{p}}^{2}\right)/n}$$

In this formula, SE_*p*_ is the standard error of the *p* ^th^ centile, SD is the standard deviation, *Z*_*p*_ is the *Z‐*score corresponding to the *p* ^th^ centile and *n* is the sample size. With a SD of 1 after standardized Box–Cox transformation, a target SE of 0.05 at the 2.5^th^ and 97.5^th^ centiles required a sample size of 1169 for all 3D‐svFMBV parameters. Utilizing the actual sample size following exclusions (*n* = 2547) and the calculated SD, the SE was found to be 0.03, affirming the adequacy of the sample size for constructing centile charts. The *bestNormalize* package in R^27^ was used to select the best method of transformation, and constant variance across gestational age was assumed.

#### Models for reference chart construction

The distribution of 3D‐svFMBV at five different locations within the uteroplacental vasculature (Figure 1, Table 1) was evaluated visually using histograms and quantile–quantile (Q–Q) plots, which were constructed using the *ggplot2* (version 3.5.0) package in R^28^. To explore changes in distribution with varying gestational age, faceted box‐and‐whiskers plots were constructed (also using *ggplot2* ^28^) for 3D‐svFMBV at each location. Consequently, we decided to explore flexible statistical methods that can account for variation in distribution across gestational age, including quantile regression (QR)^29^ and the lambda‐mu‐sigma (LMS) method^30^, instead of relying on the traditional mean and SD approach.

We used the *quantreg* package in R^31^ to perform QR, fitting linear, polynomial, natural spline and B‐spline models, with gestational age in days as the predictor. Both the Barrodale and Roberts method and penalized lasso regression were applied to estimate the quantiles.

LMS modeling was conducted utilizing the *lms* function within the *gamlss* package in R^32^. A variety of Generalized Additive Models for Location, Scale and Shape (GAMLSS) families were evaluated for 3D‐svFMBV at each target placental location to determine the most suitable distribution for the dataset. The assessed families included Box–Cox Cole and Green original (BCCGo), Box–Cox Power Exponential original (BCPEo), Box–Cox *t*‐Distribution Extended original (BCTEo), Box–Cox *t*‐Distribution (BCT), Skew Exponential Power (SEP1, SEP2, SEP3, SEP4), Sinh–Arcsinh (SHASH, SHASHo), Johnson's SU (JSU), Generalized *t*‐Distribution (GT), Log Normal (LOGNO) and Skew *t*‐Distribution Types 1–5 (ST1, ST2, ST3, ST4, ST5). The number of cycles was set to 1000, with a *k*‐value of 2 and all other LMS parameters set to default.

#### Evaluation of models

More complex models may achieve a better fit but can reduce generalizability (a paradox known as overfitting). The Akaike information criterion (AIC) was chosen as the method for model comparison because it evaluates goodness of fit while penalizing complexity^33^. Lower AIC values indicate a better balance between fit and simplicity. Charts with centile curves and raw data plots were constructed to confirm the goodness of fit of all potential models. Additionally, histograms and Q–Q plots of *Z*‐scores were constructed.

#### Analysis of excluded cases

We compared the main study cohort with the cases excluded during the third stage of QC, which were statistical outliers with respect to placental volume. Baseline characteristics were analyzed using the Wilcoxon rank‐sum test for continuous variables and Fisher's exact test for categorical variables.

## RESULTS

After exclusions, the final cohort comprised 2547 cases. The selection of this cohort is summarized in Figure S1 and the demographic and pregnancy characteristics are detailed in Table 2.

**Table 2:** Characteristics of 2547 pregnancies included in study

Variable	Value
Maternal age (years)	33.4 (30.4–36.2)
Maternal weight (kg)	66.6 (59.9–75.6)
Maternal height (cm)	166.0 (162.0–170.8)
Body mass index (kg/m^2^)	23.9 (21.5–27.2)
Ethnicity	
Black	286 (11.23)
East Asian	52 (2.04)
South Asian	164 (6.44)
White	1944 (76.33)
Mixed	101 (3.97)
Mode of conception	
Spontaneous	2368 (92.97)
Ovulation drugs	14 (0.55)
*In‐vitro* fertilization	165 (6.48)
Smoker at presentation	46 (1.81)
Parity	
Nulliparous	1209 (47.47)
Parous	1338 (52.53)
Previous PE	40 (1.57)
No previous PE	1298 (50.96)
Previous FGR	181 (7.11)
No previous FGR	1157 (45.43)
Female fetal sex	1264 (49.63)
Gestational age at birth (weeks)	39.9 (39.0–40.7)
Birth weight (g)	3440 (3195–3740)
Birth‐weight centile	51.5 (30.2–74.7)
Birth weight < 10^th^ centile	122 (4.79)

Data are given as median (interquartile range) or *n* (%). FGR, fetal growth restriction; PE, pre‐eclampsia.

### Distribution of 3D‐svFMBV values

**Figure 2:**
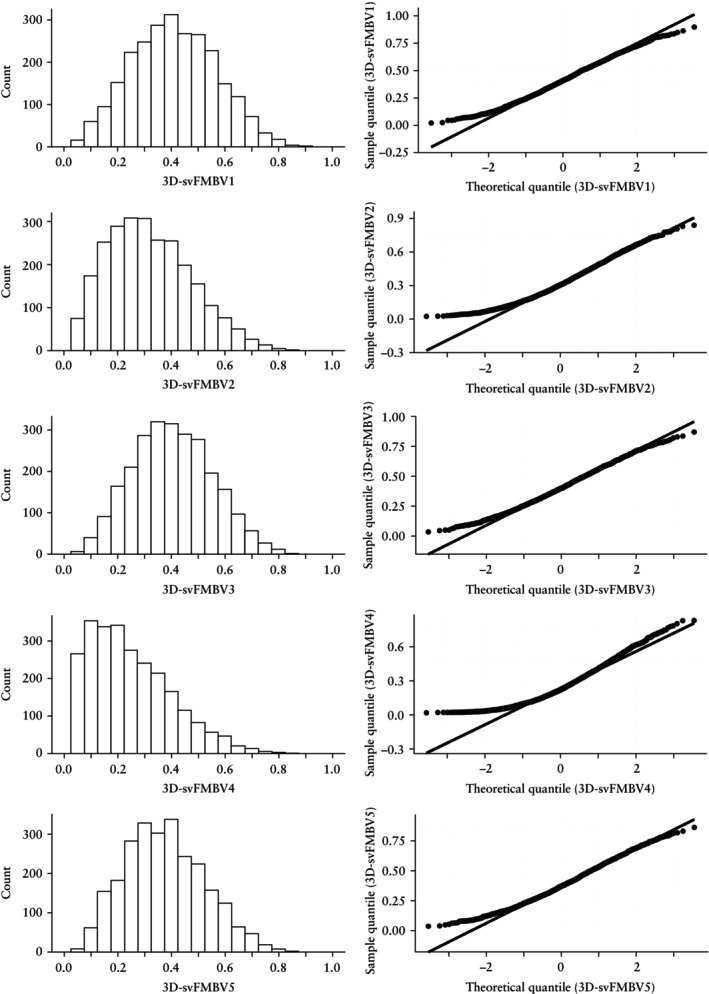
Histograms (left) and quantile−quantile plots (right) for three‐dimensional single‐vessel fractional moving blood volume (3D‐svFMBV) at five target locations within uteroplacental vasculature.

**Figure 3:**
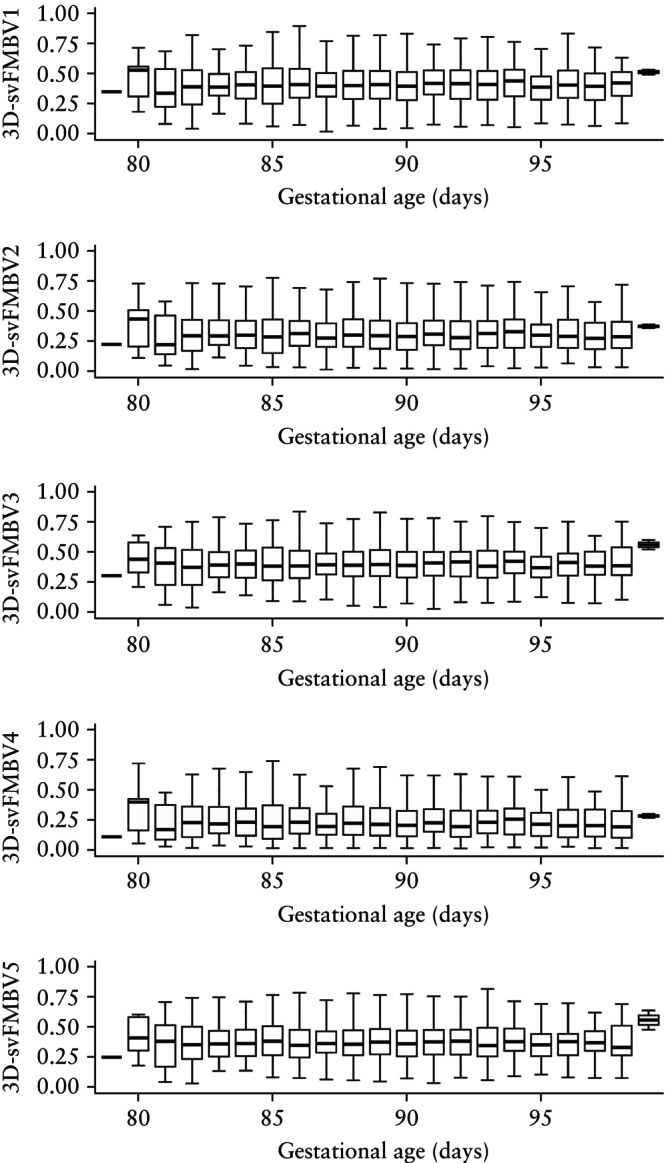
Box‐and‐whiskers plots for three‐dimensional single‐vessel fractional moving blood volume (3D‐svFMBV) at five target locations within uteroplacental vasculature, according to gestational age. Boxes with internal lines are median and interquartile range and whiskers are range.

Visual assessment of the histograms and Q–Q plots for 3D‐svFMBV1–5 revealed positive skew in all cases, which was most prominent in 3D‐svFMBV4 (Figure 2). Faceted box‐and‐whiskers plots suggest variation in the distribution of 3D‐svFMBV with gestational age (Figure 3), although the interpretation of these results was limited by low case numbers at the extremes of gestational age.

### Comparison of models

The AIC statistics for the QR models are summarized in Table 3. The AIC statistics for the LMS models with the chosen GAMLSS family are presented in Table 4. For 3D‐svFMBV1–5, the lowest AIC value was achieved using LMS modeling with the Rigby–Stasinopoulos fitting algorithm^32^. The BCPEo distribution was used, except in the case of 3D‐svFMBV2 and 3D‐svFMBV4, for which SEP3 and SEP1 were used, respectively.

**Table 3:** Akaike information criterion (AIC) assessment of quantile regression models for prediction from gestational age (in days) of three‐dimensional single‐vessel fractional moving blood volume (3D‐svFMBV) at five target locations within uteroplacental vasculature

	AIC				
Modeling approach/Method	3D‐svFMBV1	3D‐svFMBV2	3D‐svFMBV3	3D‐svFMBV4	3D‐svFMBV5
Linear					
B&R	−1806.84	−1906.37	−2162.46	−2107.87	−2168.95
PLR	−1807.03	−1907.34	−2161.76	−2109.62	−2168.01
Polynomial 2					
B&R	−1806.84	−1904.66	−2160.48	−2105.89	−2167.07
PLR	−1729.21	−1829.40	−2079.70	−2021.81	−2045.41
Polynomial 3					
B&R	−1805.04	−1902.84	−2160.17	−2105.25	−2166.45
PLR	−1550.35	−1653.30	−1923.31	−1906.62	−1909.56
Polynomial 4					
B&R	−1803.08	−1902.63	−2158.22	−2103.56	−2164.46
PLR	−1366.02	−1444.05	−1767.44	−1703.96	−1748.27
Natural splines					
B&R	−1803.79	−1902.28	−2158.90	−2103.90	−2164.72
PLR	−1754.18	−1860.15	−2110.22	−2061.37	−2102.74
B‐splines					
B&R	−1803.21	−1903.00	−2158.17	−2103.67	−2164.45
PLR	−1761.33	−1882.90	−2111.09	−2091.07	−2121.06

B&R, Barrodale and Roberts method; PLR, penalized lasso regression.

**Table 4:** Akaike information criterion (AIC) assessment of lambda‐mu‐sigma models for prediction from gestational age (in days) of three‐dimensional single‐vessel fractional moving blood volume (3D‐svFMBV) at five target locations within uteroplacental vasculature

Parameter	Selected GAMLSS family	AIC
3D‐svFMBV1	Box–Cox Power Exponential original	−2225.74
3D‐svFMBV2	Skew Exponential Power Type 3	−2464.89
3D‐svFMBV3	Box–Cox Power Exponential original	−2557.69
3D‐svFMBV4	Skew Exponential Power Type 1	−2956.33
3D‐svFMBV5	Box–Cox Power Exponential original	−2612.20

GAMLSS, Generalized Additive Models for Location, Scale and Shape.

### Final models

**Figure 4:**
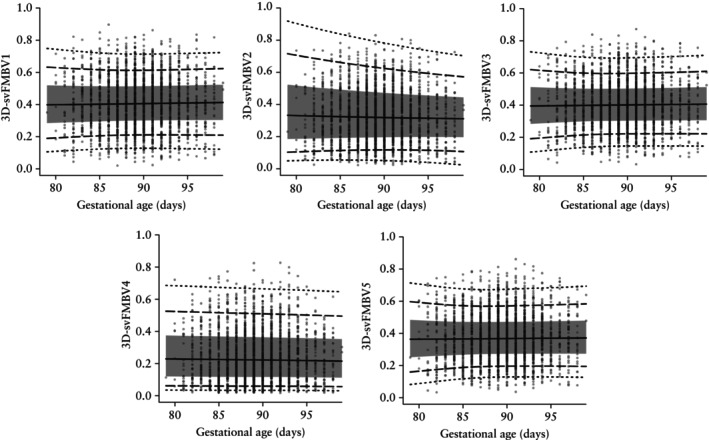
Reference charts for three‐dimensional single‐vessel fractional moving blood volume (3D‐svFMBV) at five target locations within uteroplacental vasculature. Solid line, 50^th^ centile; gray band, 25^th^–75^th^ centile; dashed lines, 10^th^ and 90^th^ centiles; dotted lines, 3^rd^ and 97^th^ centiles.

Based on their lower AIC values, the models generated by the LMS method were selected for constructing the centile charts. The resulting centile charts for 3D‐svFMBV1–5 according to gestational age are presented in Figure 4. The model summaries, including coefficients, are provided in Appendix S1. The five models are available as an R file in Appendix S2, and the R script for calculating individual centiles is available in Appendix S3.

### Evaluation of centile charts

Table 5 compares the predicted and observed centiles across nine target centiles for the five models: 1^st^, 3^rd^, 10^th^, 25^th^, 50^th^, 75^th^, 90^th^, 97^th^ and 99^th^. All models performed well, demonstrating close agreement between the centiles predicted from calibration and the observed centiles in the sample, with minimal deviation across all target centiles evaluated, including at the extremes.

**Table 5:** Percentage of cases at various centiles of first‐trimester three‐dimensional single‐vessel fractional moving blood volume (3D‐svFMBV) observed in study cohort (Obs) *vs* predicted by final model (Pred)

	3D‐svFMBV1		3D‐svFMBV2		3D‐svFMBV3		3D‐svFMBV4		3D‐svFMBV5	
Target centile	Pred (%)	Obs (%)	Pred (%)	Obs (%)	Pred (%)	Obs (%)	Pred (%)	Obs (%)	Pred (%)	Obs (%)
1^st^	1.06	1.02	1.00	1.02	0.93	1.02	0.90	1.02	0.93	1.02
3^rd^	2.56	3.02	2.64	3.02	2.81	3.02	3.74	3.02	3.12	3.02
10^th^	10.24	10.01	10.10	10.01	9.75	10.01	12.13	10.01	9.80	10.01
25^th^	24.90	25.01	25.78	25.01	25.95	25.01	26.53	25.05	25.50	25.01
50^th^	50.11	49.98	50.18	50.02	49.61	50.02	49.55	50.02	50.48	50.02
75^th^	74.73	74.99	74.50	74.99	74.46	74.99	74.59	74.99	74.75	74.99
90^th^	90.21	89.99	89.71	89.99	89.74	89.99	89.79	89.99	90.31	89.99
97^th^	97.10	96.98	97.17	96.98	97.08	96.98	97.15	96.98	97.22	96.98
99^th^	99.05	98.98	99.40	98.98	99.02	98.98	99.02	98.98	99.00	98.98

The *Z*‐scores for 3D‐svFMBV1–5, derived using each of the respective models, are presented as histograms and Q–Q plots in Figure S2. The expected normal distribution was present in all cases.

### Analysis of excluded cases

Demographic and pregnancy characteristics of the study cohort and the 15 cases excluded in the third round of QC are compared in Table S2. No statistically significant differences were found, with the exception of history of PE, which was more common among the excluded cases (2/15 (13.3%)) compared with the study cohort (40/2547 (1.6%)) (*P* < 0.001).

## DISCUSSION

This study provides reference ranges for first‐trimester 3D‐svFMBV, modeled against gestational age, at five specific locations within the uteroplacental vasculature, based on a large cohort of healthy pregnancies. We employed machine‐learning techniques, specifically the OxNNet toolkit, to achieve fully automated placental segmentation and 3D‐svFMBV calculation. Sensitivity analysis indicated no statistically significant differences between the study cohort and the 15 cases excluded during the third round of QC, except for the prevalence of PE, which was higher in the excluded cases. This is probably a statistical artifact due to the small number of excluded cases. As such, it is unlikely to impact the construction of 3D‐svFMBV centile charts, given that the overall cohort remains representative, and the models exhibit good calibration.

A wide range of 3D‐svFMBV values were observed in our cohort, consistent with previous findings^14^. In a study of 143 cases, Collins *et al*.^14^ reported FMBV values ranging from 0.01 to 0.65 at the UPI (equivalent to 3D‐svFMBV1 in our study) and < 0.01 to 0.44 in the intervillous space (equivalent to 3D‐svFMBV2 in our study). Our larger dataset enabled greater representation of values at the extremes of the normal distribution, capturing a wider spectrum of physiological variation. However, the cohort studied by Collins *et al*.^14^ included pregnancies complicated by PE and small‐for‐gestational age, for which a greater range of values might be expected in association with pathology.

To our knowledge, this is the first study to define reference values for first‐trimester placental 3D‐svFMBV. Importantly, the utility of these reference charts lies not in the binary classification of perfusion as normal or abnormal, but in their potential to contribute to multivariable predictive models for adverse outcome. Our large sample size allowed for precise estimation of 3D‐svFMBV distributions across gestational age, thereby enhancing the reliability of centile calculations, especially for extreme centiles, such as the 5^th^ and 95^th^ centiles. Our substantial sample size also ensured better representation of subgroups, which in turn improved the generalizability of the charts. Furthermore, with a greater number of data points, the impact of outliers was minimized, resulting in more robust centile charts that accurately reflect the true population distribution.

The study was further strengthened by employing the LMS method to model 3D‐svFMBV, which accounts for complex, non‐normal distributions at each 3D‐svFMBV level. Although the LMS method poses a risk of overfitting, we addressed this issue by visually inspecting the curves and utilizing the AIC statistic to evaluate model fit. This approach ensured robustness without introducing excessive complexity. Although the LMS method provides more precise modeling of 3D‐svFMBV distributions, it requires advanced mathematical knowledge to implement. To support broader use, the models and R scripts for centile calculation are provided in the supplementary material, allowing other researchers to replicate and apply the findings.

One of the primary strengths of our study is the use of the OxNNet toolkit. Fully automated placental segmentation and 3D‐svFMBV calculation enabled the creation of a larger dataset than would otherwise have been practical. The OxNNet toolkit also presents a significant advantage over semiautomated segmentation using virtual organ computer‐aided analysis (VOCAL™), which depends on geometric assumptions that may not accurately reflect the irregular shape of the placenta^34^. The shape of the placenta can vary significantly in early pregnancy, potentially rendering manual and geometric methods susceptible to errors in volume estimation^35^.

Signal normalization is critical to the accuracy of Doppler‐based perfusion estimates. The single‐vessel referencing technique employed by the OxNNet toolkit mitigates variability introduced by depth, tissue interfaces and other signal‐degrading factors. This approach is conceptually similar to the foundational work of Rubin *et al*.^7^, ^8^, although implemented differently. While the discussion of signal physics is beyond the scope of this article, the development, validation and rationale for our technique have been described previously^13^. This methodological difference compared with early work on FMBV is a potential source of variation in observations.

Temporal variability in acquisition, particularly across the cardiac cycle, is another potential source of error in Doppler imaging. While OxNNet does not incorporate cardiac gating, the averaging effect of 3D volume sweeps is expected to mitigate systolic–diastolic fluctuations.

We applied rigorous QC to both placental imaging and segmentation to ensure that the models were based on accurate placental assessments. High image quality is crucial for 3D‐svFMBV to be a reliable clinical marker. To support this, integrated QC features are being developed within the OxNNet toolkit, enabling automated, real‐time QC during image capture. This will allow sonographers to receive immediate feedback and to repeat scans if issues such as incomplete images or significant artifacts arise, thereby improving the reliability of 3D‐svFMBV measurements in clinical practice. OxNNet does not yet operate in real time, but ongoing optimization efforts aim to reduce processing time to under 60 s, making the technique feasible for clinical application.

Despite our large cohort, there were few cases at extreme gestational ages, which is a limitation for distribution analysis. Including data from earlier and later within the first trimester could improve the robustness of the model. Additionally, our study was based on a single UK center, which limits generalizability across different populations. Further research in diverse cohorts is needed. Although we excluded cases with known fetal or maternal pathology, undiagnosed conditions may have affected 3D‐svFMBV. Further research is required to understand the relationship between 3D‐svFMBV and pregnancy outcome, which can now be supported by the availability of reference ranges. External validation of these reference ranges is essential to assess diagnostic utility. This will be pursued in future studies, including the Oxford Placental Ultrasound Study (OxPLUS), which aims to evaluate the predictive value of 3D‐svFMBV *Z*‐scores and other vascular markers in pathological pregnancies.

OxNNet is designed to be vendor‐agnostic through the use of NIfTI format conversion, allowing compatibility across different ultrasound platforms. While this mitigates concerns about system variability, further multicenter studies using diverse imaging systems are necessary to confirm generalizability and reproducibility.

In conclusion, this study provides novel reference ranges for first‐trimester placental 3D‐svFMBV at five locations within the uteroplacental vasculature, leveraging advanced machine‐learning techniques for automated segmentation and 3D‐svFMBV calculation. The large sample size enhances the reliability of centile charts, while the use of the LMS method ensures robust modeling of 3D‐svFMBV distributions. Despite certain limitations, such as cohort generalizability and the rarity of cases at extreme gestational ages, these findings offer a valuable foundation for future research into placental function and pregnancy outcome. The availability of reference ranges and supporting materials should facilitate further validation and clinical application, improving early pregnancy assessment and advancing obstetric care.

## Supporting information


**Table S1** Ultrasound machine settings.

**Table S2** Sensitivity analysis of demographic and pregnancy characteristics in study cohort *vs* cases excluded in third round of quality control.

**Figure S1** Flowchart summarizing selection of study cohort. APS, antiphospholipid syndrome; FirstPLUS, First‐trimester Placental Ultrasound; QC, quality control; SLE, systemic lupus erythematosus.

**Figure S2** Histograms (left) and quantile−quantile plots (right) showing distribution of *Z*‐scores for three‐dimensional single‐vessel fractional moving blood volume (3D‐svFMBV) at five target locations within uteroplacental vasculature.

**Appendix S1** Model summaries.

**Appendix S2** RData file containing models.

**Appendix S3** R script for calculation of individual centiles.

## Data Availability

The data that support the findings of this study are available on request from the corresponding author. The data are not publicly available due to privacy or ethical restrictions.
