# Supplementary material for: Reference charts for first‐trimester placental three‐dimensional fractional moving blood volume derived using OxNNet
Source: Ultrasound Obstet Gynecol. 2026 Jan 7;67(2):191–200. doi: 10.1002/uog.70161 (PMC12865523; doi:10.1002/uog.70161)
Supplement: Supplementary file 3 — Figure S1 Flowchart summarizing selection of study cohort. APS, antiphospholipid syndrome; FirstPLUS, First‐trimester Placental Ultrasound; QC, quality control; SLE, systemic lupus erythematosus. [file UOG-67-191-s001.pptx]

## Slide 1
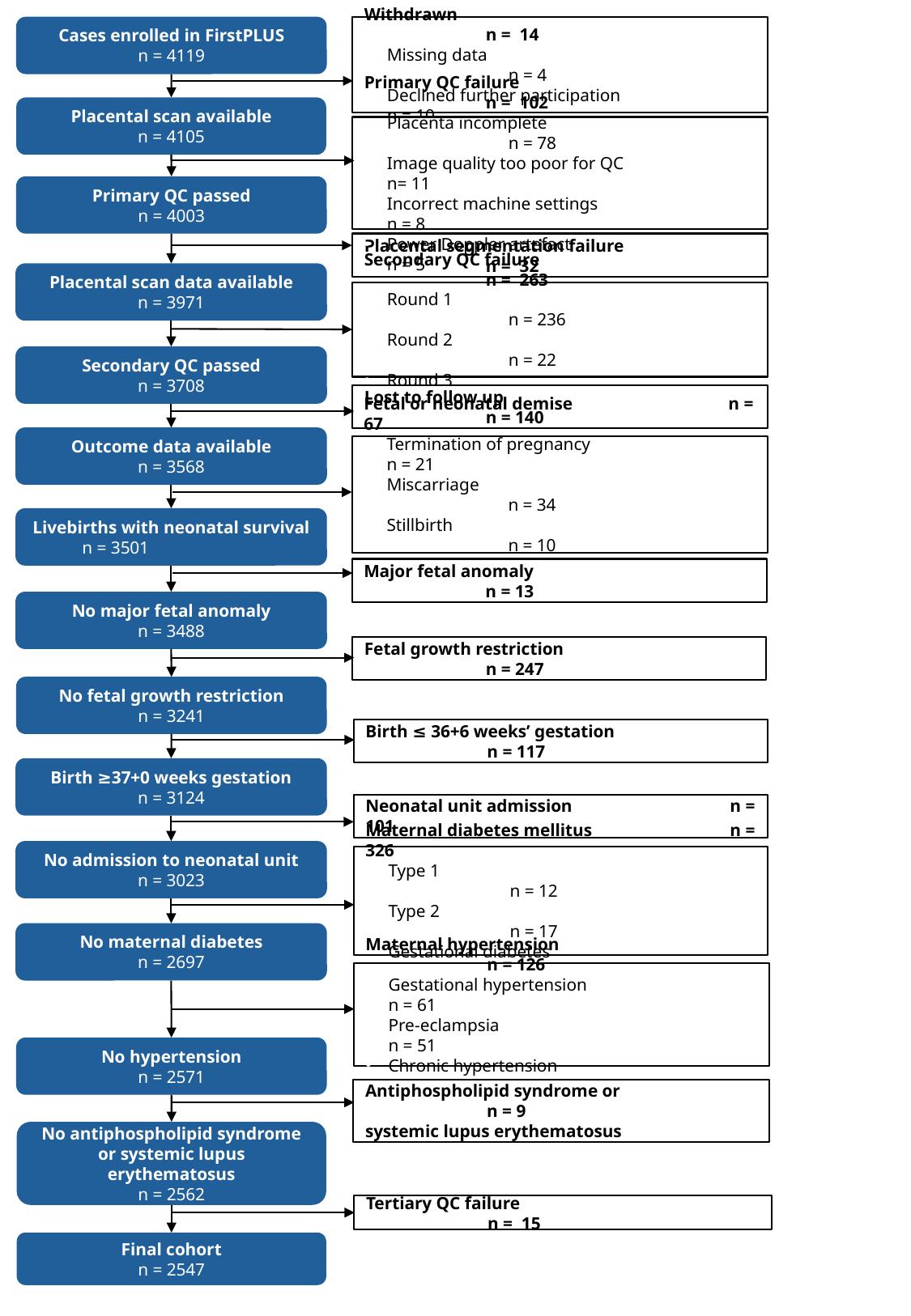

Cases enrolled in FirstPLUS
n = 4119
Withdrawn				n = 14
Missing data				n = 4
Declined further participation		n = 10
Placental scan available
n = 4105
Primary QC failure			n = 102
Placenta incomplete			n = 78
Image quality too poor for QC 		n= 11
Incorrect machine settings		n = 8
Power Doppler artefact		n = 5
Primary QC passed
n = 4003
Placental segmentation failure		n = 32
Placental scan data available
n = 3971
Secondary QC failure			n = 263
Round 1				n = 236
Round 2				n = 22
Round 3				n = 5
Secondary QC passed
n = 3708
Lost to follow up 			n = 140
Outcome data available
n = 3568
Fetal or neonatal demise		n = 67
Termination of pregnancy		n = 21
Miscarriage				n = 34
Stillbirth				n = 10
Neonatal death			n = 2
Livebirths with neonatal survival
n = 3501
Major fetal anomaly			n = 13
No major fetal anomaly
n = 3488
Fetal growth restriction			n = 247
No fetal growth restriction
n = 3241
Birth ≤ 36+6 weeks’ gestation		n = 117
Birth ≥37+0 weeks gestation
n = 3124
Neonatal unit admission		n = 101
No admission to neonatal unit
n = 3023
Maternal diabetes mellitus		n = 326
Type 1				n = 12
Type 2				n = 17
Gestational diabetes			n = 297
No maternal diabetes
n = 2697
Maternal hypertension			n = 126
Gestational hypertension		n = 61
Pre-eclampsia			n = 51
Chronic hypertension			n = 14
No hypertension
n = 2571
Antiphospholipid syndrome or		n = 9
systemic lupus erythematosus
No antiphospholipid syndrome or systemic lupus erythematosus
n = 2562
Tertiary QC failure			n = 15
Final cohort
n = 2547
