# Supplementary figures and images for: Reference charts for first‐trimester placental three‐dimensional fractional moving blood volume derived using OxNNet
Source: Ultrasound Obstet Gynecol. 2026 Jan 7;67(2):191–200. doi: 10.1002/uog.70161 (PMC12865523; doi:10.1002/uog.70161)

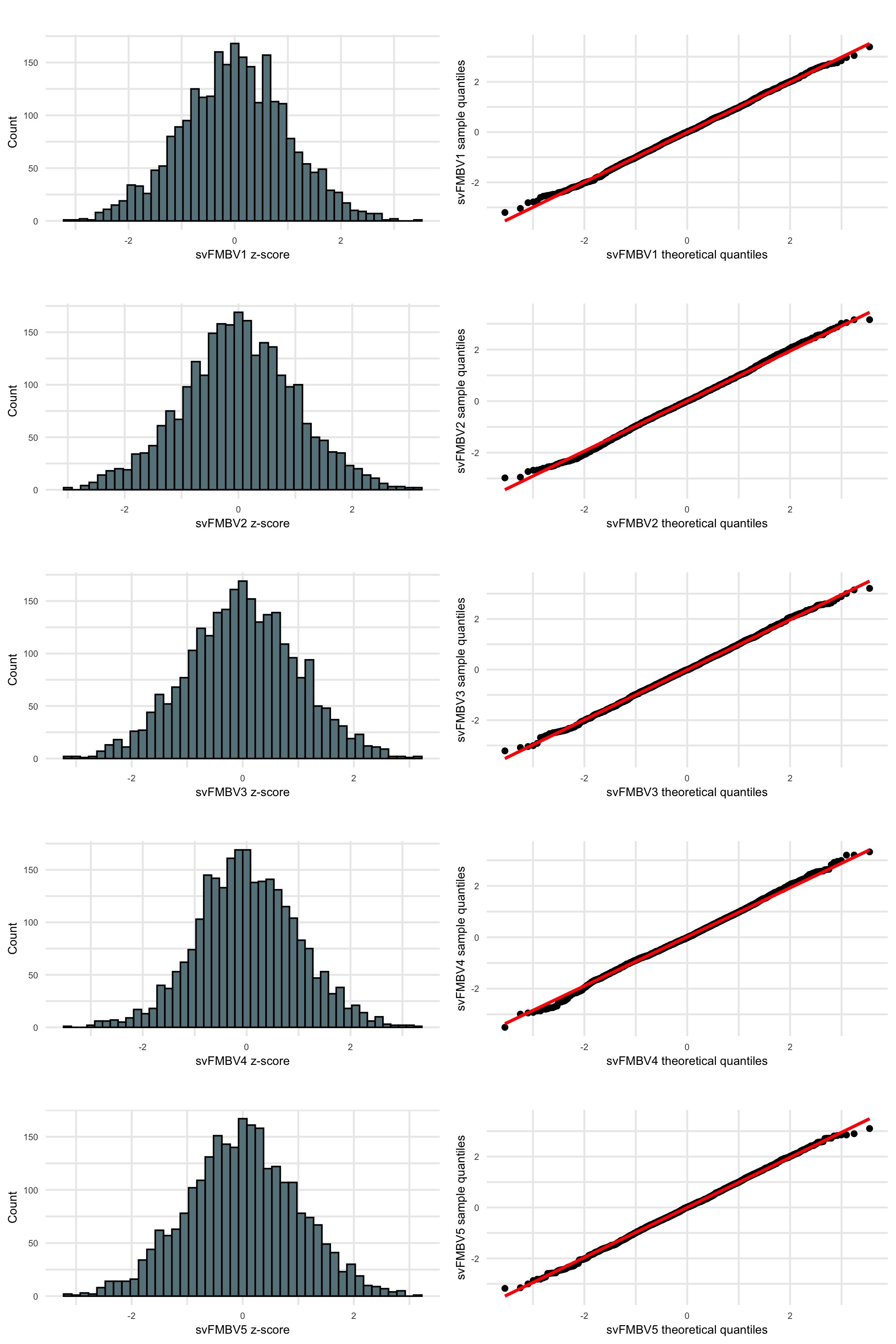

Supplement: Supplementary file 4 — Figure S2 Histograms (left) and quantile−quantile plots (right) showing distribution of Z‐scores for three‐dimensional single‐vessel fractional moving blood volume (3D‐svFMBV) at five target locations within uteroplacental vasculature. [file UOG-67-191-s002.jpeg]
