# Supplementary material for: Reference charts for first‐trimester placental three‐dimensional fractional moving blood volume derived using OxNNet
Source: Ultrasound Obstet Gynecol. 2026 Jan 7;67(2):191–200. doi: 10.1002/uog.70161 (PMC12865523; doi:10.1002/uog.70161)
Supplement: Supplementary file 5 — Appendix S1 Model summaries. [file UOG-67-191-s006.docx]

**Appendix S1** Model summaries

**Model for FMBV1**

******************************************************************

Family: c("BCPEo", "Box-Cox Power Exponential-orig.")

Call:

gamlss(formula = fmbv1 ~ pb(GA), sigma.formula = ~pb(GA), nu.formula = ~pb(GA),

, tau.formula = ~pb(GA), familfmbv1 = "BCPEo", data = dataTEMP,

, n.cfmbv1c = 1000)

Fitting method: RS()

------------------------------------------------------------------

Mu link function: log

Mu Coefficients:

Estimate Std. Error t value Pr(>|t|)

(Intercept) -1.057092 0.164133 -6.440 1.42e-10

pb(x, df = mu.df) 0.001733 0.001836 0.944 0.345

(Intercept) ***

pb(x, df = mu.df)

---

Signif. codes:

0 ‘***’ 0.001 ‘**’ 0.01 ‘*’ 0.05 ‘.’ 0.1 ‘ ’ 1

------------------------------------------------------------------

Sigma link function: log

Sigma Coefficients:

Estimate Std. Error t value

(Intercept) -0.607600 0.298083 -2.038

pb(x, df = sigma.df) -0.003686 0.003343 -1.103

Pr(>|t|)

(Intercept) 0.0416 *

pb(x, df = sigma.df) 0.2702

---

Signif. codes:

0 ‘***’ 0.001 ‘**’ 0.01 ‘*’ 0.05 ‘.’ 0.1 ‘ ’ 1

------------------------------------------------------------------

Nu link function: identity

Nu Coefficients:

Estimate Std. Error t value Pr(>|t|)

(Intercept) 0.269555 0.812374 0.332 0.740

pb(x, df = nu.df) 0.006785 0.009119 0.744 0.457

------------------------------------------------------------------

Tau link function: log

Tau Coefficients:

Estimate Std. Error t value Pr(>|t|)

(Intercept) 2.53849 1.17104 2.168 0.0303

pb(x, df = tau.df) -0.01751 0.01308 -1.339 0.1808

(Intercept) *

pb(x, df = tau.df)

---

Signif. codes:

0 ‘***’ 0.001 ‘**’ 0.01 ‘*’ 0.05 ‘.’ 0.1 ‘ ’ 1

------------------------------------------------------------------

NOTE: Additive smoothing terms exist in the formulas:

i) Std. Error for smoothers are for the linear effect only.

ii) Std. Error for the linear terms may not be reliable.

------------------------------------------------------------------

No. of observations in the fit: 2547

Degrees of Freedom for the fit: 8.961042

Residual Deg. of Freedom: 2538.039

at cycle: 11

Global Deviance: -2243.657

AIC: -2225.735

SBC: -2173.378

******************************************************************

**Model for FMBV2**

******************************************************************

Family: c("SEP3", "skew exponential power type 3")

Call:

gamlss(formula = fmbv2 ~ pb(GA), sigma.formula = ~pb(GA), nu.formula = ~pb(GA),

, tau.formula = ~pb(GA), familfmbv2 = "SEP3", data = dataTEMP,

n.cfmbv2c = 1000)

Fitting method: RS()

------------------------------------------------------------------

Mu link function: identity

Mu Coefficients:

Estimate Std. Error t value

(Intercept) -0.3565128 0.0582331 -6.122

pb(x, df = mu.df) 0.0061101 0.0006547 9.332

Pr(>|t|)

(Intercept) 1.07e-09 ***

pb(x, df = mu.df) < 2e-16 ***

---

Signif. codes:

0 ‘***’ 0.001 ‘**’ 0.01 ‘*’ 0.05 ‘.’ 0.1 ‘ ’ 1

------------------------------------------------------------------

Sigma link function: log

Sigma Coefficients:

Estimate Std. Error t value

(Intercept) -2.381665 0.296559 -8.031

pb(x, df = sigma.df) 0.006252 0.003322 1.882

Pr(>|t|)

(Intercept) 1.46e-15 ***

pb(x, df = sigma.df) 0.06 .

---

Signif. codes:

0 ‘***’ 0.001 ‘**’ 0.01 ‘*’ 0.05 ‘.’ 0.1 ‘ ’ 1

------------------------------------------------------------------

Nu link function: log

Nu Coefficients:

Estimate Std. Error t value Pr(>|t|)

(Intercept) 3.360625 0.262674 12.79 <2e-16

pb(x, df = nu.df) -0.031075 0.002939 -10.57 <2e-16

(Intercept) ***

pb(x, df = nu.df) ***

---

Signif. codes:

0 ‘***’ 0.001 ‘**’ 0.01 ‘*’ 0.05 ‘.’ 0.1 ‘ ’ 1

------------------------------------------------------------------

Tau link function: log

Tau Coefficients:

Estimate Std. Error t value

(Intercept) 2.219961 0.537062 4.134

pb(x, df = tau.df) -0.013065 0.005987 -2.182

Pr(>|t|)

(Intercept) 3.69e-05 ***

pb(x, df = tau.df) 0.0292 *

---

Signif. codes:

0 ‘***’ 0.001 ‘**’ 0.01 ‘*’ 0.05 ‘.’ 0.1 ‘ ’ 1

------------------------------------------------------------------

NOTE: Additive smoothing terms exist in the formulas:

i) Std. Error for smoothers are for the linear effect only.

ii) Std. Error for the linear terms may not be reliable.

------------------------------------------------------------------

No. of observations in the fit: 2547

Degrees of Freedom for the fit: 8.141301

Residual Deg. of Freedom: 2538.859

at cycle: 101

Global Deviance: -2481.177

AIC: -2464.894

SBC: -2417.327

******************************************************************

**Model for FMBV3**

******************************************************************

Family: c("BCPEo", "Box-Cox Power Exponential-orig.")

Call:

gamlss(formula = fmbv3 ~ pb(GA), sigma.formula = ~pb(GA), nu.formula = ~pb(GA),

, tau.formula = ~pb(GA), familfmbv3 = "BCPEo", data = dataTEMP,

, n.cfmbv3c = 1000)

Fitting method: RS()

------------------------------------------------------------------

Mu link function: log

Mu Coefficients:

Estimate Std. Error t value Pr(>|t|)

(Intercept) -1.048429 0.160633 -6.527 8.08e-11

pb(x, df = mu.df) 0.001485 0.001799 0.826 0.409

(Intercept) ***

pb(x, df = mu.df)

---

Signif. codes:

0 ‘***’ 0.001 ‘**’ 0.01 ‘*’ 0.05 ‘.’ 0.1 ‘ ’ 1

------------------------------------------------------------------

Sigma link function: log

Sigma Coefficients:

Estimate Std. Error t value

(Intercept) -0.638557 0.303151 -2.106

pb(x, df = sigma.df) -0.003939 0.003405 -1.157

Pr(>|t|)

(Intercept) 0.0353 *

pb(x, df = sigma.df) 0.2475

---

Signif. codes:

0 ‘***’ 0.001 ‘**’ 0.01 ‘*’ 0.05 ‘.’ 0.1 ‘ ’ 1

------------------------------------------------------------------

Nu link function: identity

Nu Coefficients:

Estimate Std. Error t value Pr(>|t|)

(Intercept) 0.962774 0.865048 1.113 0.266

pb(x, df = nu.df) -0.001645 0.009725 -0.169 0.866

------------------------------------------------------------------

Tau link function: log

Tau Coefficients:

Estimate Std. Error t value Pr(>|t|)

(Intercept) 3.44546 1.15824 2.975 0.00296

pb(x, df = tau.df) -0.02810 0.01293 -2.173 0.02985

(Intercept) **

pb(x, df = tau.df) *

---

Signif. codes:

0 ‘***’ 0.001 ‘**’ 0.01 ‘*’ 0.05 ‘.’ 0.1 ‘ ’ 1

------------------------------------------------------------------

NOTE: Additive smoothing terms exist in the formulas:

i) Std. Error for smoothers are for the linear effect only.

ii) Std. Error for the linear terms may not be reliable.

------------------------------------------------------------------

No. of observations in the fit: 2547

Degrees of Freedom for the fit: 9.218249

Residual Deg. of Freedom: 2537.782

at cycle: 11

Global Deviance: -2576.122

AIC: -2557.686

SBC: -2503.827

******************************************************************

**Model for FMBV4**

******************************************************************

Family: c("SEP1", "Skew exponential power (Azzalini type 1)")

Call:

gamlss(formula = fmbv4 ~ pb(GA), sigma.formula = ~pb(GA), nu.formula = ~pb(GA),

, tau.formula = ~pb(GA), familfmbv4 = "SEP1", data = dataTEMP,

n.cfmbv4c = 1000)

Fitting method: RS()

------------------------------------------------------------------

Mu link function: identity

Mu Coefficients:

Estimate Std. Error t value

(Intercept) 3.058e-02 2.053e-02 1.490

pb(x, df = mu.df) -7.334e-05 2.285e-04 -0.321

Pr(>|t|)

(Intercept) 0.136

pb(x, df = mu.df) 0.748

------------------------------------------------------------------

Sigma link function: log

Sigma Coefficients:

Estimate Std. Error t value

(Intercept) -1.1530200 0.3095555 -3.725

pb(x, df = sigma.df) -0.0004301 0.0034533 -0.125

Pr(>|t|)

(Intercept) 0.0002 ***

pb(x, df = sigma.df) 0.9009

---

Signif. codes:

0 ‘***’ 0.001 ‘**’ 0.01 ‘*’ 0.05 ‘.’ 0.1 ‘ ’ 1

------------------------------------------------------------------

Nu link function: identity

Nu Coefficients:

Estimate Std. Error t value Pr(>|t|)

(Intercept) -9.3846 551.5037 -0.017 0.986

pb(x, df = nu.df) 0.8484 6.0796 0.140 0.889

------------------------------------------------------------------

Tau link function: log

Tau Coefficients:

Estimate Std. Error t value

(Intercept) 1.224565 0.787066 1.556

pb(x, df = tau.df) -0.003039 0.008742 -0.348

Pr(>|t|)

(Intercept) 0.120

pb(x, df = tau.df) 0.728

------------------------------------------------------------------

NOTE: Additive smoothing terms exist in the formulas:

i) Std. Error for smoothers are for the linear effect only.

ii) Std. Error for the linear terms may not be reliable.

------------------------------------------------------------------

No. of observations in the fit: 2547

Degrees of Freedom for the fit: 8.660604

Residual Deg. of Freedom: 2538.339

at cycle: 54

Global Deviance: -2973.649

AIC: -2956.327

SBC: -2905.726

******************************************************************

**Model for FMBV5**

******************************************************************

Family: c("BCPEo", "Box-Cox Power Exponential-orig.")

Call:

gamlss(formula = fmbv5 ~ pb(GA), sigma.formula = ~pb(GA), nu.formula = ~pb(GA),

, tau.formula = ~pb(GA), familfmbv5 = "BCPEo", data = dataTEMP,

, n.cfmbv5c = 1000)

Fitting method: RS()

------------------------------------------------------------------

Mu link function: log

Mu Coefficients:

Estimate Std. Error t value Pr(>|t|)

(Intercept) -1.113788 0.173858 -6.406 1.77e-10

pb(x, df = mu.df) 0.001231 0.001949 0.631 0.528

(Intercept) ***

pb(x, df = mu.df)

---

Signif. codes:

0 ‘***’ 0.001 ‘**’ 0.01 ‘*’ 0.05 ‘.’ 0.1 ‘ ’ 1

------------------------------------------------------------------

Sigma link function: log

Sigma Coefficients:

Estimate Std. Error t value

(Intercept) -0.540237 0.294562 -1.834

pb(x, df = sigma.df) -0.004086 0.003314 -1.233

Pr(>|t|)

(Intercept) 0.0668 .

pb(x, df = sigma.df) 0.2177

---

Signif. codes:

0 ‘***’ 0.001 ‘**’ 0.01 ‘*’ 0.05 ‘.’ 0.1 ‘ ’ 1

------------------------------------------------------------------

Nu link function: identity

Nu Coefficients:

Estimate Std. Error t value Pr(>|t|)

(Intercept) 1.246319 0.769574 1.619 0.105

pb(x, df = nu.df) -0.006221 0.008665 -0.718 0.473

------------------------------------------------------------------

Tau link function: log

Tau Coefficients:

Estimate Std. Error t value Pr(>|t|)

(Intercept) 4.25430 1.17086 3.634 0.000285

pb(x, df = tau.df) -0.03696 0.01306 -2.830 0.004693

(Intercept) ***

pb(x, df = tau.df) **

---

Signif. codes:

0 ‘***’ 0.001 ‘**’ 0.01 ‘*’ 0.05 ‘.’ 0.1 ‘ ’ 1

------------------------------------------------------------------

NOTE: Additive smoothing terms exist in the formulas:

i) Std. Error for smoothers are for the linear effect only.

ii) Std. Error for the linear terms may not be reliable.

------------------------------------------------------------------

No. of observations in the fit: 2547

Degrees of Freedom for the fit: 10.31922

Residual Deg. of Freedom: 2536.681

at cycle: 20

Global Deviance: -2632.843

AIC: -2612.204

SBC: -2551.912

******************************************************************
